# Topical tranexamic acid in elderly patients with femoral neck fractures treated with hemiarthroplasty: efficacy and safety? - a case-control study

**DOI:** 10.1186/s12891-019-2615-z

**Published:** 2019-05-17

**Authors:** Dae-Kyung Kwak, Chul-Young Jang, Dae-Hwan Kim, Sang-Hyun Rhyu, Ji-Hyo Hwang, Je-Hyun Yoo

**Affiliations:** 1 Department of Orthopaedic Surgery, Hallym University Sacred Heart Hospital, Hallym University College of Medicine, 896 Pyeongchon-dong, Dongan-gu, Anyang, 14068 South Korea; 2 Department of Orthopaedic Surgery, Kangnam Sacred Heart Hospital, Hallym University College of Medicine, Seoul, South Korea

**Keywords:** Hip, Femoral neck fracture, Bipolar hemiarthroplasty, Tranexamic acid, Topical administration

## Abstract

**Background:**

Perioperative blood management is an important issue in the treatment of elderly patients at an increased risk of postoperative complications. Accordingly, tranexamic acid (TXA) is widely administered to reduce blood loss and transfusion requirements. In this case-control study, the effect of topical TXA on the outcomes of elderly patients with femoral neck fractures after hemiarthroplasty was evaluated.

**Methods:**

This study enrolled elderly patients (age ≥ 70 years) who underwent cementless bipolar hemiarthroplasty for femoral neck fractures between January 2015 and January 2017. The study group comprised 72 patients who received TXA via topical administration during surgery. After propensity matching, the control group comprised 72 patients who did not receive topical TXA. The perioperative and postoperative parameters of the two groups were compared.

**Results:**

The estimated blood loss, vacuum tube drainage, and total transfusion volume were significantly lower in the study group than the control (*p* = 0.024, 0.003, and 0.019, respectively). Despite a lack of significant intergroup differences in the lengths of ICU and hospital stays; rates of ICU admission, venous thromboembolism, delirium, and readmission; and rates of in-hospital and 1-year mortality, the incidence of postoperative medical complications was significantly lower in the study group (*p* = 0.003).

**Conclusion:**

Topical TXA administration appears to be a simple and effective option for reducing blood loss, transfusion requirements, and medical complications after hemiarthroplasty in elderly patients with femoral neck fractures.

## Background

The incidence of osteoporotic hip fracture continues to increase among elderly patients worldwide in accordance with increasing life expectancies and surgical advancement [1]. Femoral neck fracture is a common type of hip fracture in aging populations and is most effectively managed with hemiarthroplasty, which is reported to have relatively good outcomes [2]. Although recent advances in perioperative management and rehabilitation have reduced the incidence of mortality after hip fracture [3, 4], the majority of elderly patients have multiple comorbidities and poor physical conditions. For example, anemia is highly prevalent (39–69%) among elderly patients undergoing hip fracture surgery [5–7]. This condition is likely to increase cardiac demand and cause potential tissue hypoxia, which could potentially decrease physical performance and thereby impede early rehabilitation [8]. Anemia has been also associated with several complications such as infection, immunologic reaction, and morbidity, and placed a high burden on the affected patients [9–11]. Therefore, careful attention should be paid to perioperative bleeding control.

Various routine blood management strategies have been implemented to reduce perioperative blood loss in elderly patients [12, 13]. One such strategy uses the drug, tranexamic acid (TXA), a synthetic amino acid derivative. This effective antifibrinolytic agent competitively inhibits the conversion of plasminogen to plasmin to stabilize clotting [14]. Despite controversies regarding the use of TXA, numerous studies have confirmed that this drug effectively reduces blood loss when administered either intravenously (IV) or orally [15–17]. Kang et al. [18] and Sanz et al. [19] reported that the topical administration of TXA is an effective and safe method for reducing postoperative blood loss and transfusion requirements in patients receiving hip arthroplasty.

Despite the importance of bleeding control after hemiarthroplasty for femoral neck fractures, especially in elderly patients, the current literature contains little information about the effects of topical TXA on surgical outcomes after hemiarthroplasty in this population. This case-control study was conducted to evaluate whether the topical administration of TXA would reduce perioperative blood losses and transfusion requirements and determine the effects of this simple procedure on the post-hemiarthroplasty outcomes of elderly patients with femoral neck fractures.

## Methods

This retrospective cohort study was approved by the institutional review board of our hospital. Patients who met the following inclusion criteria were included: (1) age ≥ 70 years at the time of injury, (2) acute femoral neck fracture, (3) subsequent treatment with cementless bipolar hemiarthroplasty between January 2015 and January 2017, and (4) available postoperative follow-up records for at least 12 months. Two hundred twenty-six patients who met the inclusion criteria, were finally identified. Notably, patients who underwent hemiarthroplasty before April 2016 were not administered topical TXA during surgery, whereas patients treated during and after April 2016 were administered this drug to reduce blood loss and postoperative complications. Because the addition of the topical TXA procedure depended only on the date of surgery, no patient selection bias was present. The 226 patients included 72 and 154 patients who did (study group) and did not receive topical TXA during hemiarthroplasty.

All operations were performed using the same implant via the posterolateral approach by a single senior surgeon who specialized in hip and trauma surgery. All femoral components were inserted in a press-fit manner. After implantation and massive irrigation, 1 g TXA (500 mg/A, Shinpoong Pharm. Co. Ltd., Ansan, South Korea) that had been prepared in a 10-cc syringe for topical intraarticular injection, was injected into the joint capsule and soft tissue around the hip joint just before wound closure. Procedurally, the two groups only differed in terms of the intraarticular injection of TXA. In all cases, the short external rotators were preserved during surgery and the posterior capsule was routinely repaired. Additionally, a vacuum drain was inserted in the hip joint and removed 48 h after surgery in all patients.

After surgery, all patients in both groups received a combined chemical thromboprophylaxis for 10–14 days and mechanical thromboprophylaxis until discharge (about 2 weeks). Low-molecular-weight heparin was used as a chemoprophylactic agent, and an intermittent pneumatic compression device and graduated compression stockings were used as mechanical prophylaxes. According to our institutional protocol, patients were instructed to walk under partial weight-bearing conditions with an assistive device (walker or cane) from the second postoperative day to 4–6 weeks postoperatively. Routine follow-up visits were scheduled at 6 weeks and 3, 6, 9, and 12 months postoperatively, and annually thereafter. Patients who did not return for regularly scheduled visits or their families were contacted by telephone.

The following data were collected from medical records. Demographic data at the time of surgery included the age, gender, body mass index (BMI), bone mineral density (BMD), comorbidities, and American Society of Anesthesiologists (ASA) score. Perioperative parameters included the time to operation; preoperative anticoagulant medication administered for comorbidities; preoperative levels of hemoglobin (Hb), hematocrit (Hct), albumin, and cholesterol; operation time, anesthesia method; postoperative changes in Hb and Hct levels; volumes of vacuum tube drainage, transfusion, and estimated blood loss (EBL); lengths of intensive care unit (ICU) and hospital stays; and the incidence of ICU admission. Postoperative data included the rates of venous thromboembolism, delirium, and readmission; medical and surgical complications; and rates of in-hospital and 1-year mortality; and functional outcomes assessed by the Koval score and Harris hip score (HHS) 6 months postoperatively for patients alive.

Patients received transfusions of packed red blood cells when the Hb level decreased below 8 g/dL or at higher levels if a poor general condition, palpitations, dizziness, or pallor was observed.

The EBL was calculated according to the Gross formula, as shown below.

Prediction of blood volume [20]:

$$ \mathrm{Male}:604+0.0003668\times {\left[\mathrm{Height}\ \left(\mathrm{cm}\right)\right]}^3+32.2\times \mathrm{weight}\ \left(\mathrm{kg}\right) $$

$$ \mathrm{Female}:183+0.000356\times {\left[\mathrm{Height}\ \left(\mathrm{cm}\right)\right]}^3+33\times \mathrm{weight}\ \left(\mathrm{kg}\right) $$

Estimated blood loss (EBL) calculation method [21]:

$$ \mathrm{Estimated}\ \mathrm{blood}\ \mathrm{loss}=\mathrm{blood}\ \mathrm{volume}\times \left({\mathrm{Hct}}_{\mathrm{preoperative}}-{\mathrm{Hct}}_{\mathrm{day}\ 5\ \mathrm{postoperative}}\right)+\mathrm{ml}\ \mathrm{of}\ \mathrm{transfused}\ \mathrm{RBC} $$

For calculation of EBL, we used Hct levels just before surgery and at postoperative day (POD) 5 and the volume of transfused red blood cells (RBC) during this period. Actually, all transfusion in our cohort was performed during surgery and within POD 3.

**Fig. 1:**
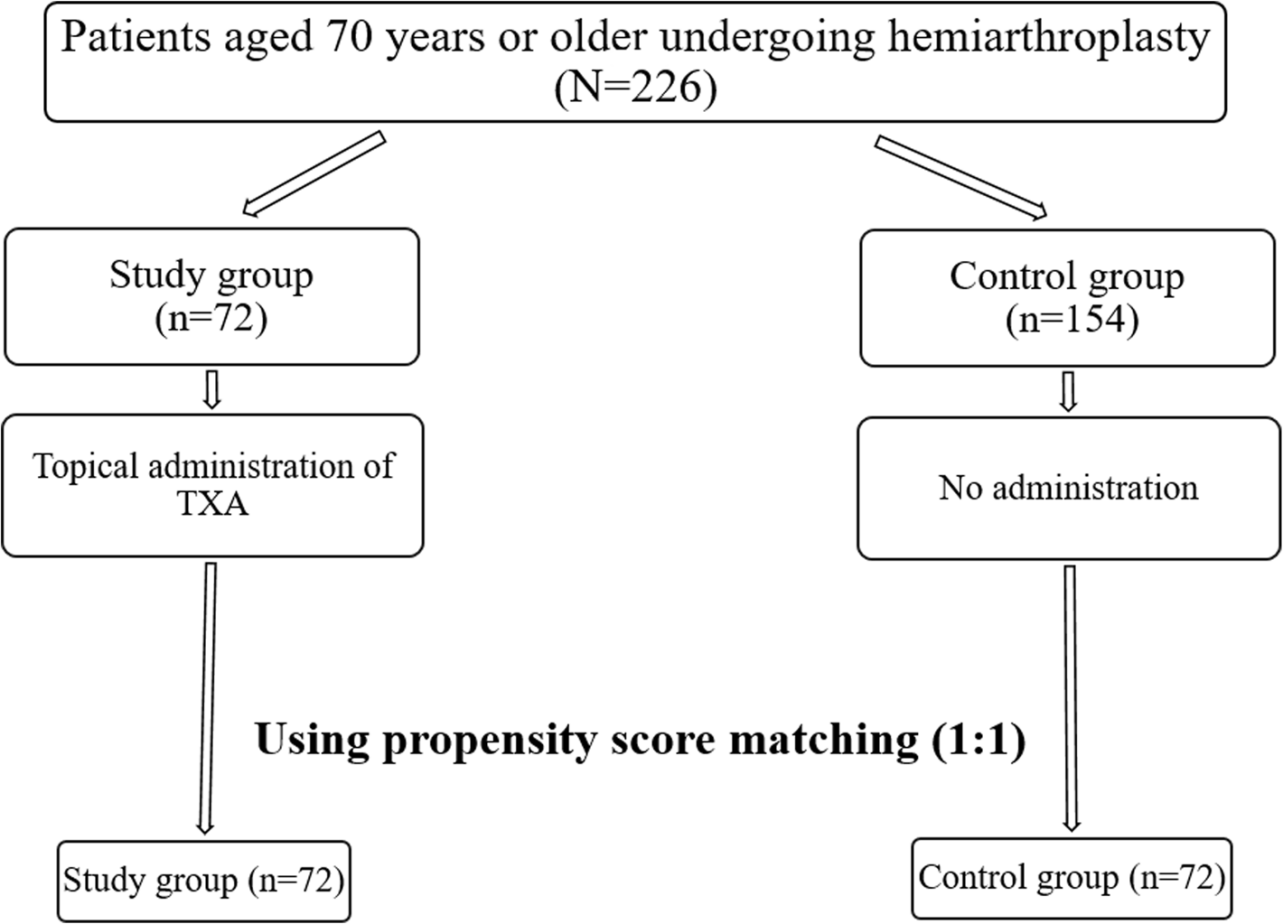
Flowchart demonstrating patient selection

**Fig. 2:**
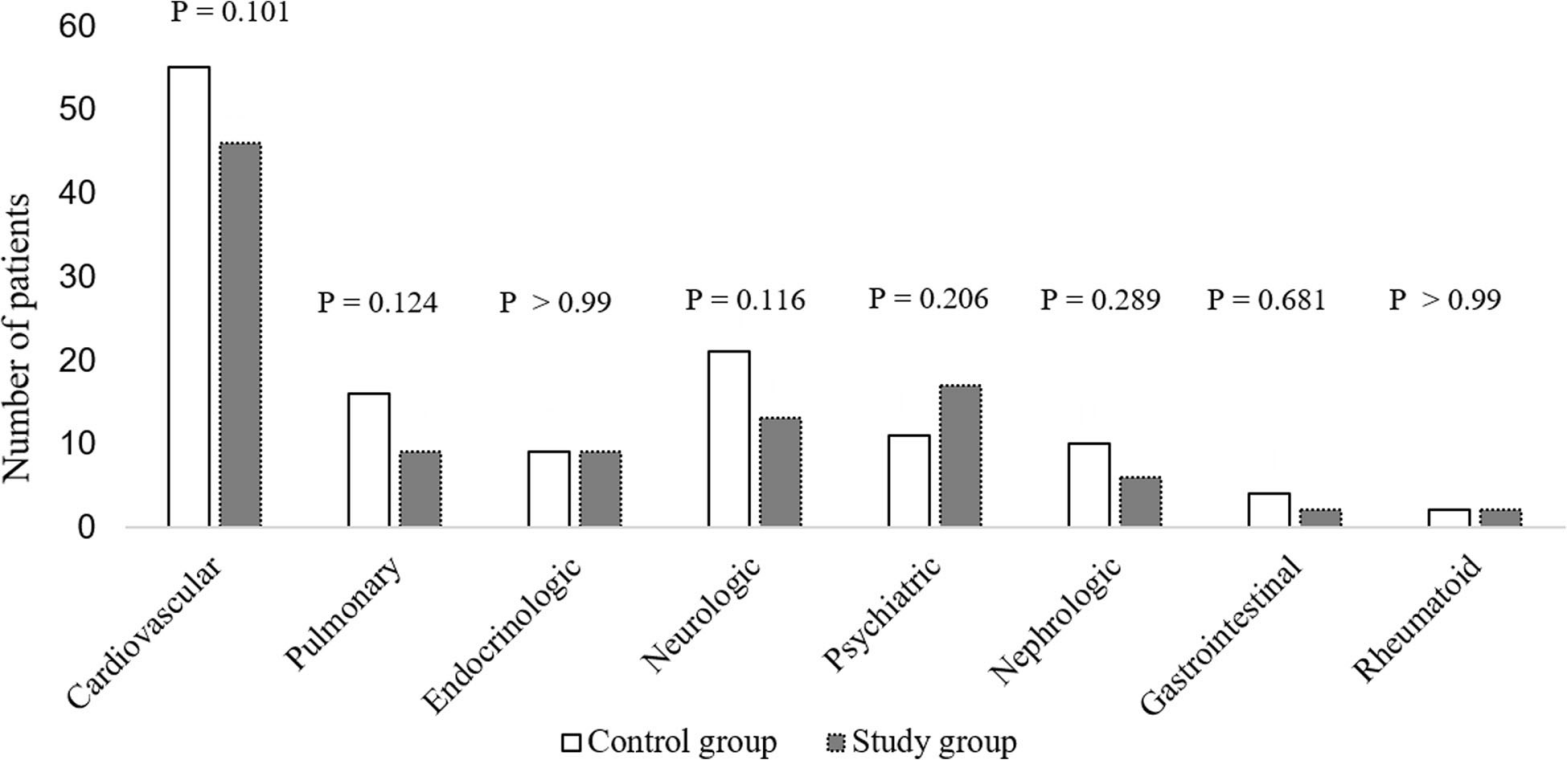
Comorbid medical diseases between the two groups

Propensity score matching was performed prior to analysis to minimize selection bias. The maximum difference in the propensity score of any matched pair was set to 0.1 for the variables of age, gender, BMI, ASA score, and time to surgery. As the number of patients (*n* = 154) not receiving topical TXA far exceeded that of the study group (*n* = 72), all patients in the latter group were included in the final analysis and matched with a patient that did not receive topical TXA. Hereafter, these 72 latter patients comprise the control group (Fig. 1). Due to the propensity matching process, the groups did not differ significantly in terms of age, gender, BMI, ASA score, and time to operation (Table 1). Moreover, the study and control groups did not differ significantly in terms of preoperative anticoagulant medication administration; BMD; preoperative levels of Hb, Hct, albumin, and cholesterol; and comorbid medical diseases (Fig. 2).

**Table 1:** Demographic and preoperative data in both groups

	Control group (*n* = 72)	Study group (*n* = 72)	*p*-value
Age	80.9 ± 6.2	80.1 ± 5.3	0.361
Gender (Male: Female)	18: 54	18: 54	> 0.99
Body mass index (kg/m^2^)	21.8 ± 4.2	22.3 ± 3.5	0.414
Time to operation (days)	2.8 ± 2.2	3.0 ± 3.9	0.656
ASA score			0.166
II	1	0	
III	69	65	
IV	2	7	
Preoperative anticoagulant medication	19 (26.4%)	24 (33.3%)	0.363
Bone mineral density (T-score)	−3.0 ± 1.2	−2.8 ± 1.0	0.344
Preoperative levels			
Hemoglobin (g/dL)	12.1 ± 1.5	11.5 ± 1.7	0.063
Hematocrit (%)	36.1 ± 4.5	35.1 ± 3.6	0.141
Albumin (g/dL)	3.7 ± 0.4	3.7 ± 0.4	0.923
Cholesterol (mg/dL)	164.1 ± 37.6	158.7 ± 42.6	0.416

Continuous variables are presented as mean ± standard deviation

*ASA* American society of anesthesiologists

Once the two groups contained the same numbers of matched patients (*n* = 72), further statistical analyses were conducted. Independent t-tests were used to compare numerical data, which are expressed as means±standard deviations (SDs) and ranges. The chi-square and Fisher’s exact tests were used to compare categorical data. For all analyses, a *p*-value of < 0.05 was considered to indicate statistical significance. IBM SPSS, version 24.0 (IBM Corp., Armonk, NY) was used for all statistical analyses in this study.

## Results

No significant intergroup differences were observed in the operation time; anesthesia method; immediate postoperative and POD 1 and 5 levels of Hb and Hct; lengths of ICU and hospital stays; and the rate of ICU admission (Table 2).

**Table 2:** Comparison of perioperative data between the two groups

	Control group (n = 72)	Study group (n = 72)	*p*-value
Operation time (min)	76.1 ± 12.5	78.4 ± 22.3	0.349
Anesthesia (general: spinal)	63:9	63: 9	> 0.99
Immediate postoperative Hb (g/dL)	10.6 ± 1.8	10.6 ± 1.2	0.991
POD 1 Hb (g/dL)	9.9 ± 1.2	10.2 ± 1.1	0.138
POD 1 Hct (%)	29.5 ± 3.3	30.1 ± 4.5	0.147
POD 5 Hb (g/dL)	10.0 ± 1.0	9.5 ± 1.0	0.152
POD 5 Hct (%)	30.3 ± 3.2	28.9 ± 4.7	0.210
Transfusion rate (%)	65.2 (47/72)	36.1 (26/72)	**0.002**
Transfusion rate (%) in Hb > 8 g/dL	31.9 (15/47)	30.7 (8/26)	0.431
Hospital stay (days)	23.4 ± 9.8	21.2 ± 6.8	0.184
ICU admission (%)	48.6 (35/72)	38.9 (28/72)	0.240
ICU stay (days)	3.4 ± 4.2	2.3 ± 0.9	0.161

Continuous variables are presented as mean ± standard deviation

*Hb* Hemoglobin, *POD* Postoperative day, *Hct* Hematocrit, *ICU* Intensive care unit

Bold indicates a statistically significant value (< 0.05)

**Fig. 3:**
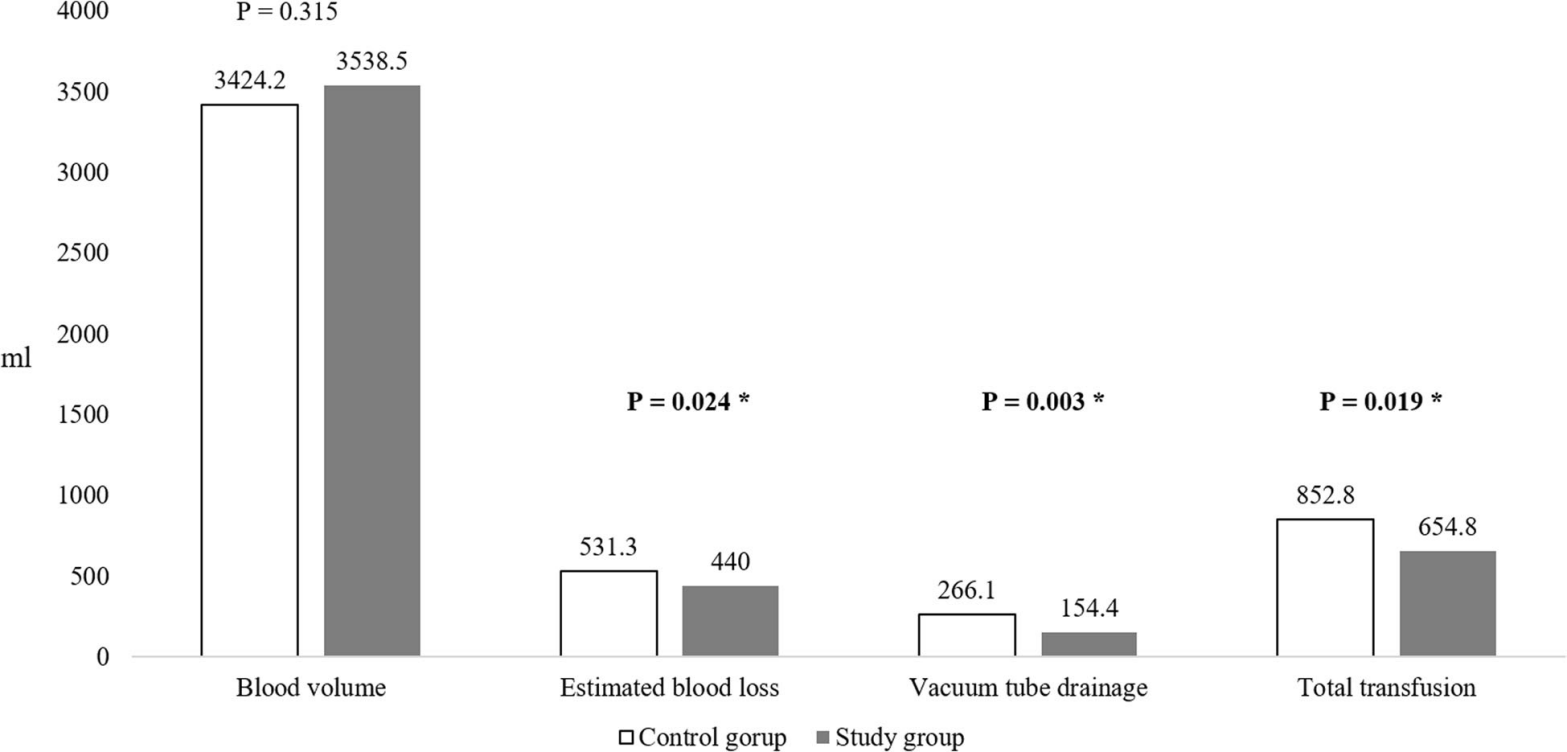
Comparison of perioperative blood loss between the two groups

However, the groups differed significantly in terms of the total EBL and vacuum tube volumes, which were 531.3 ± 21.2 and 266.1 ± 56.3 mL in the control group and 440.0 ± 13.7 and 154.4 ± 87.1 mL in the study group, respectively (*p* = 0.024 and 0.003, respectively). Furthermore, the total blood transfusion volume was significantly greater in the control group relative to the study group (852.8 ± 72.9 vs. 654.8 ± 44.4 mL, *p* = 0.019) (Fig. 3). The transfusion rate was higher in the control group (65.2%) than the study group (36.1%) (*p* = 0.002). Meanwhile, there was no significant difference in the transfusion rate at Hb > 8 g/dL perioperatively between the control and study group (31.9% vs. 30.7%, *p* = 0.431) (Table 2).

The two groups did not differ in terms of the incidences of deep vein thrombosis, pulmonary embolism, delirium, and readmission or in terms of the in-hospital and 1-year mortality rates. However, the control group had a significantly higher frequency of medical complications, compared to the study group (56.9% vs. 31.9%, *p* = 0.003). However, the groups did not differ significantly in terms of the frequency of surgical complications, such as wound infection, periprosthetic joint infection, and dislocation (Table 3). In terms of functional outcome assessed 6 months after surgery, the mean Koval score was 3.3 and 3.0 in the control group and the study, respectively; the mean HHS was 64.2 in the control group and 65.3 in the study group. There were no significant differences in Koval walking ability and clinical outcome between the two groups (Table 4).

**Table 3:** Comparison of postoperative complications between the two groups

	Control group (*n* = 72)	Study group (*n* = 72)	*p*-value
DVT	1 (1.4%)	3 (4.2%)	0.620
PE	0	1 (1.4%)	0.920
Delirium	23 (31.9%)	19 (26.4%)	0.463
Readmission	2 (2.8%)	1 (1.4%)	> 0.99
In-hospital mortality	0	1 (1.4%)	> 0.99
1-year mortality	4 (5.4%)	4 (5.4%)	> 0.99
Medical complications	41 (56.9%)	23 (31.9%)	**0.003**
Cardiovascular	7	3	
Pulmonary	11	12	
Cerebrovascular	3	0	
Nephrologic	7	1	
Urologic	10	5	
Gastrointestinal	3	2	
Surgical complications	1	0	> 0.99
Dislocation	1	0	
PJI	0	0	
Wound infection	0	0	

*DVT* Deep vein thrombosis, *PE* Pulmonary embolism, *PJI* Periprosthetic joint injection

Bold indicates a statistically significant value (< 0.05)

**Table 4:** Comparison of functional outcomes between the two groups 6 months after surgery

	Control group (*n* = 69)	Study group (*n* = 68)	*p*-value
Koval score	3.3 ± 2.1	3.0 ± 1.7	0.452
Harris hip score	64.2 ± 13.6	65.3 ± 18.3	0.871

Continuous variables are presented as mean ± standard deviation

Bold indicates a statistically significant value (< 0.05)

## Discussion

Our study revealed that the intraoperative topical administration of TXA significantly reduced perioperative blood loss and blood transfusion volumes, as well as postoperative medical complications, in elderly patients with femoral neck fractures who underwent hemiarthroplasty. These findings suggest that this simple and cost-efficient procedure is highly effective in elderly patients undergoing hemiarthroplasty for femoral neck fractures.

TXA has been widely used to reduce blood losses and transfusion requirements following hip arthroplasty, and has been shown to confer these benefits when established in IV, topical, and oral forms [22–24]. Several studies have demonstrated that blood transfusion results in a longer hospital stay and increased morbidity and mortality, and have identified it as an independent risk factor for periprosthetic joint infection [25–27]. Another study demonstrated that a restrictive transfusion policy leads to increased cardiovascular events and increased mortality rates [28]. However, transfusion, regardless of the amount of transfusion, is associated with an increased long-term mortality after hip fracture surgery [5]. Therefore, it is important to reduce perioperative blood loss and transfusion requirement after hip fracture surgery, especially in elderly patients with comorbidities. However, the current literature contains little information about the effects of TXA on surgical outcomes after arthroplasty in elderly patients with hip fracture.

Recent systematic reviews and meta-analyses have reported that the topical application of TXA to the joint during surgery might be effective [29–31]. Furthermore, this administration route was shown to reduce systemic effects, compared to oral or IV TXA administration, but was not found to increase the risk of thromboembolic events [31, 32]. Therefore, we conducted this case-control study to evaluate the efficacy and safety of topical TXA in terms of surgical outcomes after hemiarthroplasty in elderly patients with femoral neck fracture.

In our study, topical TXA significantly reduced perioperative blood loss, vacuum drainage, and rate and total amount of blood transfusion in our cohort, although it did not affect the change of Hb and Hct levels, the lengths of ICU and hospital stays or the rates of in-hospital and 1-year mortalities. In terms of blood loss, we measured total blood loss from operation to postoperative day 5 using the Gross formula (Mercuriali’s formula) based on the volume of transfused RBC and the change of hematocrit during the meanwhile and compared between the two groups. Accordingly, we believe that total blood loss estimated in our study was relatively accurate and that topical TXA injection reduced postoperative bleeding although there might be no difference in intraoperative bleeding between the two groups. Topical TXA injected into surrounding soft tissue, capsule, and muscles might reduce postoperative bleeding leaked out from these structures. Topical TXA significantly reduced the transfusion rate in the case group in spite of only small differences in the change of Hb and Hct levels between the two groups. We believe that small difference of Hb and Hct at postoperative day 1 and 5 between the two groups was caused by compensation by subsequent more transfusion due to more blood loss during early postoperative period in the control group.

Consistent with our findings, Tuttle et al. [33] reported that topical TXA reduced transfusion rates and costs and increased the frequency of hospital discharge to home rather than to a subacute nursing facility among patients undergoing primary hip and knee arthroplasty, but noted no significant difference in the length of hospital stay. The current study also revealed that transfusion rate was significantly lower in the study group than control group. However, the rate of transfusion in this study is relatively higher than other studies reporting 6 to 26.2% [34, 35]. In our hospital, anesthesiologists empirically tend to decide intraoperative transfusion considering patients’ comorbidities and conditions. Therefore, more elderly patients at high risk of complication due to comorbidities tended to receive intraoperative transfusion even at Hb > 8 g/dL. More intraoperative transfusions performed by anesthesiologists might increases total transfusion rate in our cohort. Also, some patients received postoperative transfusion at Hb > 8 g/dL according to the recommendation of specialists considering patients’ comorbidities and conditions, postoperatively. For these reasons, the transfusion rate in our study was relatively high compared to other studies. However, there was no significant difference in transfusion rate at Hb > 8 g/dL perioperatively between the two group. Similar portion of patients with these comorbidities and medical conditions in both groups would have shown similar rate of transfusion at Hb > 8 g/dL perioperatively between the two groups. Finally, these findings mean that more patients in the control group not receiving topical TXA injection received transfusion at Hb ≤8 g/dL due to more perioperative blood loss.

As noted, there have been no previous studies reporting on the effects of topical TXA, including postoperative complications and mortality, in fragile elderly patients. Our investigation revealed that topical TXA administration reduced the incidence of postoperative medical complications after hemiarthroplasty in these patients but had no significant effects on surgical complications and mortality. We therefore believe that reductions in postoperative blood loss and transfusion requirement mediated by topical TXA, directly or indirectly reduced postoperative medical complications in these fragile patients. It would be difficult to turn out that these medical complications were the result of an entirely increased total blood loss. However, the patients receiving more transfusion due to more perioperative blood loss would be delayed in ambulation and rehabilitation, and less active than patients not receiving postoperative transfusion. These conditions may have influenced the occurrence of medical complications such as cardiovascular, urologic and nephrologic problems, directly or indirectly.

Several authors have reported that the topical administration of TXA reduces blood loss and transfusion rates in patients undergoing primary hip and knee arthroplasty [32, 36, 37] and that the topical administration allows the maximum application of TXA directly to the surgical site while preventing potential systemic side effects. However, no consistent guidelines have been established regarding the dose and method of topical TXA administration. Regarding the determination of TXA dosage, Alshryda et al. performed a meta-analysis of 14 randomized controlled trials regarding the topical administration of TXA in total hip and knee arthroplasty and reported that the doses ranged from 250 to 3000 mg [31]. In another meta-analysis by Chen et al. [30], the doses of topical TXA during total hip arthroplasty varied from 0.5 to 5 g. In our study, we applied a relatively lower TXA dose of 1 g, given our concerns regarding the safety of this drug in elderly patients and less invasive nature of hemiarthroplasty relative to total hip arthroplasty, particularly as the effect of topical TXA is not dose-dependent [31, 38]. Regarding the topical application method, Konig et al. [32] bathed the joint in 20 mL of a TXA solution, while Yue et al. [22] applied gauze soaked in TXA solution to the acetabulum and femoral canal based on the method of Konig et al. [32]. We opted not to use the gauze-packing method because of concerns of surgical prolongation. Moreover, Kang et al. [18] administered a TXA solution into the joint through a drainage tube immediately after wound closure. However, this method might reduce the drug effect because TXA might have leaked from joint through the drain tube. Therefore, we directly injected TXA into the joint capsule and periarticular soft tissue.

Regarding strengths of this study, it was the first to evaluate the effects of topical TXA, including postoperative complications and mortality, in fragile elderly patients (≥ 70 years) undergoing bipolar hemiarthroplasty for femoral neck fracture. Both the study and control groups underwent procedures performed by the same surgeon via the same approach. Accordingly, topical TXA administration was the sole independent variable. Furthermore, the analysis was strengthened by propensity matching in terms of demographic data, ASA score, and time to operation between the two groups. Finally, our evaluation of the amount of total blood loss, which was based on the Hct levels and Gross formula, was more accurate than evaluations based on clinical calculation involving blood-soaked gauzes, suction bottles, and vacuum drains.

However, this study also had the following limitations. Despite the use of propensity score matching, this was a retrospective study with a small number of patients in each group. Furthermore, the incidence of thromboembolism was not assessed accurately, as only patients with clinical symptoms were subjected to diagnostic tests such as ultrasonography or 3-dimensionalCT-angiography.

## Conclusions

The current study showed that topical TXA administration significantly reduced the rate and total amount of blood transfusion, blood loss, and medical complications after hemiarthroplasty in elderly patients with femoral neck fractures. These results suggest that topical TXA is a simple and effective option for perioperative blood management in elderly patients undergoing hemiarthroplasty. However, further studies with larger sample sizes are needed to confirm the efficacy and safety of topical TXA in this population.

## Abbreviations

ASA: American Society of Anesthesiologists
BMD: Bone mineral density
BMI: Body mass index
EBL: Estimated blood loss
Hb: Hemoglobin
Hct: Hematocrit
HHS: Harris hip score
ICU: Intensive care unit
IV: Interavenous
POD: Postoperative day
RBC: Red blood cell
TXA: Tranexamic acid
